# Characterization of neuropsychiatric symptoms in a group of individuals with manifest or pre-motor Huntington’s disease in Medellín, Colombia

**DOI:** 10.1192/j.eurpsy.2022.1148

**Published:** 2022-09-01

**Authors:** D. Vasquez, M. Agudelo, C. Gomez, D. Aguillon, J. Quintero, S. Rassi, M. Zuluaga, D. Pineda, O. Buritica, F. Lopera

**Affiliations:** 1 Universidad de Antioquia, Grupo De Neurociencias De Antioquia, Medellin, Colombia; 2 Universidad de Antioquia, Neurología, Medellin, Colombia; 3 Universidad de Antioquia, Psiquiatría, Medellin, Colombia

**Keywords:** Huntington’s disease, Motor symptoms, Neuropsychiatric symptoms, Chorea

## Abstract

**Introduction:**

Huntington’s disease (HD) is a rare (1-9/100 000), inherited disease characterized by an elongated CAG repeat on chromosome 4p, leading to a degeneration of neurons. Also, psychiatric symptoms are very common in the early stage and may appear before motor symptoms.

**Objectives:**

To characterize neuropsychiatric symptoms in a group of individuals with manifest or pre-motor Huntington’s disease in Medellín, Colombia.

**Methods:**

Data obtained from clinical records of individuals with HD (motor-manifest or pre-motor with triplets count) evaluated for ENROLL-HD project in the Group of Neuroscience of Antioquia. We explored variables related to substances abuse, neuropsychiatric symptoms, the respective age of onset, sex, and triplet count when available.

**Results:**

Twenty-six (53%) were women, 8% had a familiar history of psychotic illness in a first-degree relative and 88% presented motor symptoms. Also, 59% had a history of depression, 53% irritability, 57% aggressiveness, 34% apathy, 29% perseverative/obsessive behavior, 14% psychosis, and 30% mild cognitive impairment. Ten individuals (20%) had motor without neuropsychiatric symptoms. Also, thirty-seven individuals (76%) presented motor and neuropsychiatric symptoms; of these, 41% had neuropsychiatric symptoms before motor symptoms. No psychiatric symptoms were associated with the use of alcohol, cigarettes, or drugs of abuse.

**Conclusions:**

Neuropsychiatric symptoms are highly prevalent among individuals with HD and studies oriented to create relevant knowledge for the development of advice oriented to people with this disease are necessary.

**Disclosure:**

No significant relationships.

